# Endodontic Management of a Mandibular First Molar With Radix Entomolaris: A Clinical Case Report

**DOI:** 10.7759/cureus.82342

**Published:** 2025-04-16

**Authors:** Mohamed Rahhali, Majid Sakout

**Affiliations:** 1 Conservative Dentistry and Endodontics, Faculty of Dentistry, Mohammed V University, Mohammed V Military Hospital, Rabat, MAR

**Keywords:** anatomical variation, endodontic treatment, mandibular molar, radix entomolaris, root canal system

## Abstract

Successful endodontic treatment depends on a comprehensive understanding of root canal anatomy and its possible variations. Radix entomolaris (RE) is a rare anatomical variant characterized by the presence of a supernumerary distolingual root, most frequently found in mandibular first molars. This case report describes the clinical and radiographic identification of an RE in the mandibular first molar of a 42-year-old male patient presenting with symptomatic irreversible pulpitis. The additional root was identified during access cavity preparation, necessitating modification of the access outline to enable detection of all canal orifices. In this case, the RE exhibited no mesiodistal curvature and did not conform to established morphological classifications. Given the clinical challenges associated with such anatomical anomalies, heightened vigilance is essential to prevent iatrogenic complications and ensure treatment success. Early recognition and appropriate adaptation of endodontic procedures are critical for long-term tooth preservation and favorable outcomes.

## Introduction

The primary objective of endodontic treatment is to eliminate microbial contamination from the root canal system and prevent reinfection. This is achieved through meticulous biomechanical cleaning of the pulp space followed by three-dimensional obturation using an appropriate sealing material. A thorough understanding of root canal anatomy, including its possible variations, is essential for successful clinical outcomes. Among these anatomical variations, the radix entomolaris (RE) represents a supernumerary third root typically located distolingually in mandibular molars. It may occur in first, second, or third molars, though it is least common in the second.

From an epidemiological perspective, the prevalence of RE is relatively low, ranging from 3% to 7% in Caucasian, African, Eurasian, and Indian populations [1-3]. In contrast, it is significantly higher (5% to 30%) among individuals with Asian traits, including Chinese, Inuit, and Native American groups [4-6]. Understanding not only the frequency but also the morphologic variations of RE is essential for appropriate clinical management, as these can affect both diagnosis and treatment planning.

The diagnosis of RE is primarily radiographic, supported by clinical indicators such as eccentric canal entry points. Differential diagnosis should consider other conditions that mimic RE, such as the radix paramolaris located buccally, a second distolingual canal within a single root, or a lingually curved distal root, all of which require careful radiographic interpretation to avoid confusion.

This article presents a clinical case of a mandibular first molar with RE, highlighting the diagnostic considerations, the differential diagnosis, and the access cavity modifications required to ensure safe and effective endodontic treatment.

## Case presentation

A 42-year-old male patient presented to the Department of Conservative Dentistry and Endodontics at the Mohammed V Military Teaching Hospital in Rabat, Morocco, with a chief complaint of severe pain in the left mandibular posterior region that began the previous night. The pain was continuous, exacerbated by hot food and beverages, and lasted approximately two hours.

**Figure 1:**
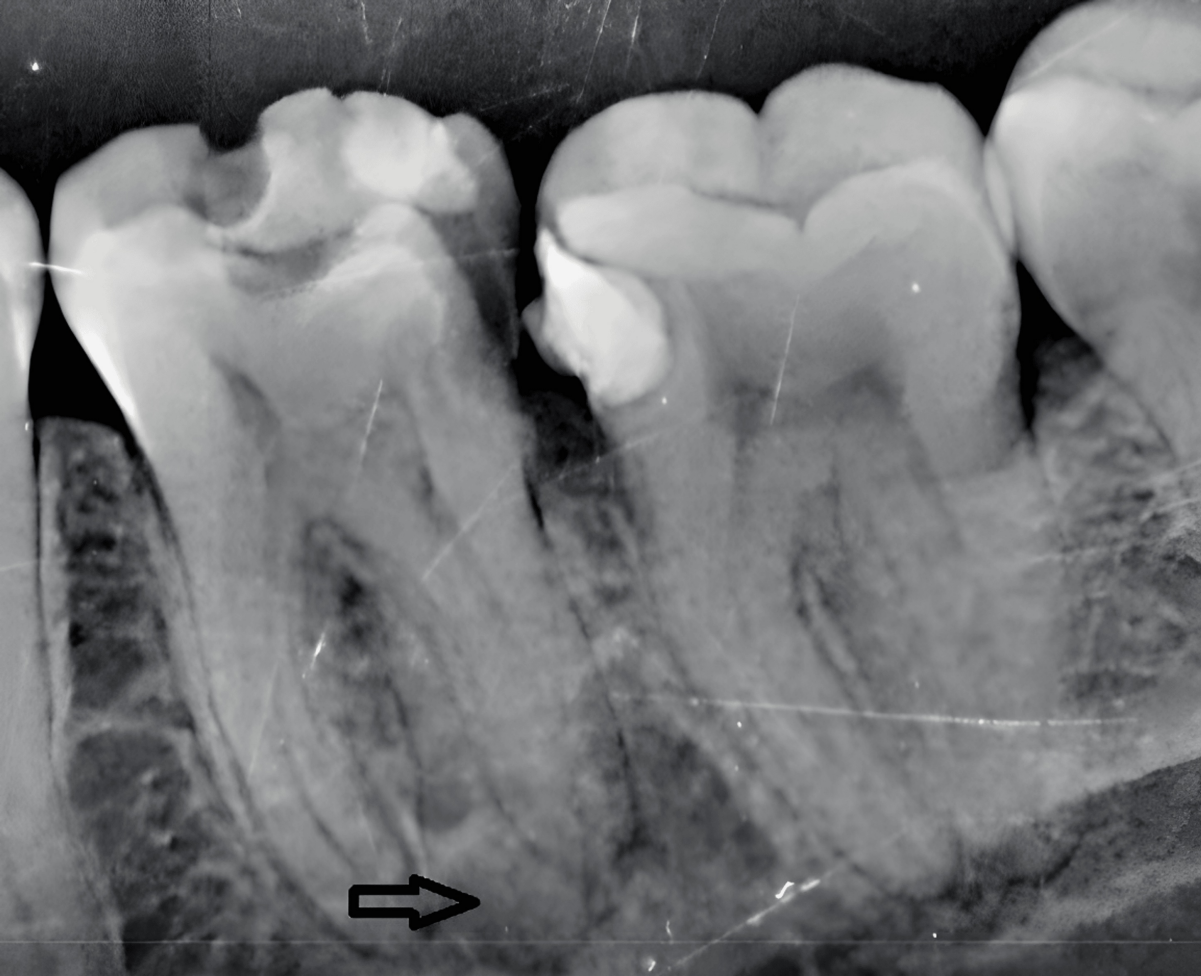
Preoperative periapical radiograph of tooth #36 showing recurrent caries and suspected additional root.

Clinical examination revealed secondary caries associated with a previously restored left mandibular first molar. The periapical radiograph showed a defective occluso-distal composite restoration with recurrent caries approaching the pulp and suggested the possible presence of an additional root (Figure 1).

The diagnosis of initial acute periodontitis was established. Following administration of regional anesthesia via an inferior alveolar nerve block, an access cavity was prepared using an Endo-Z bur (Dentsply Maillefer, Ballaigues, Switzerland). The first distal canal was located on the buccal aspect, raising suspicion of a second distal canal. The access cavity was modified from a triangular to a trapezoidal shape to enhance visualization and access to a possible fourth canal.

**Figure 2:**
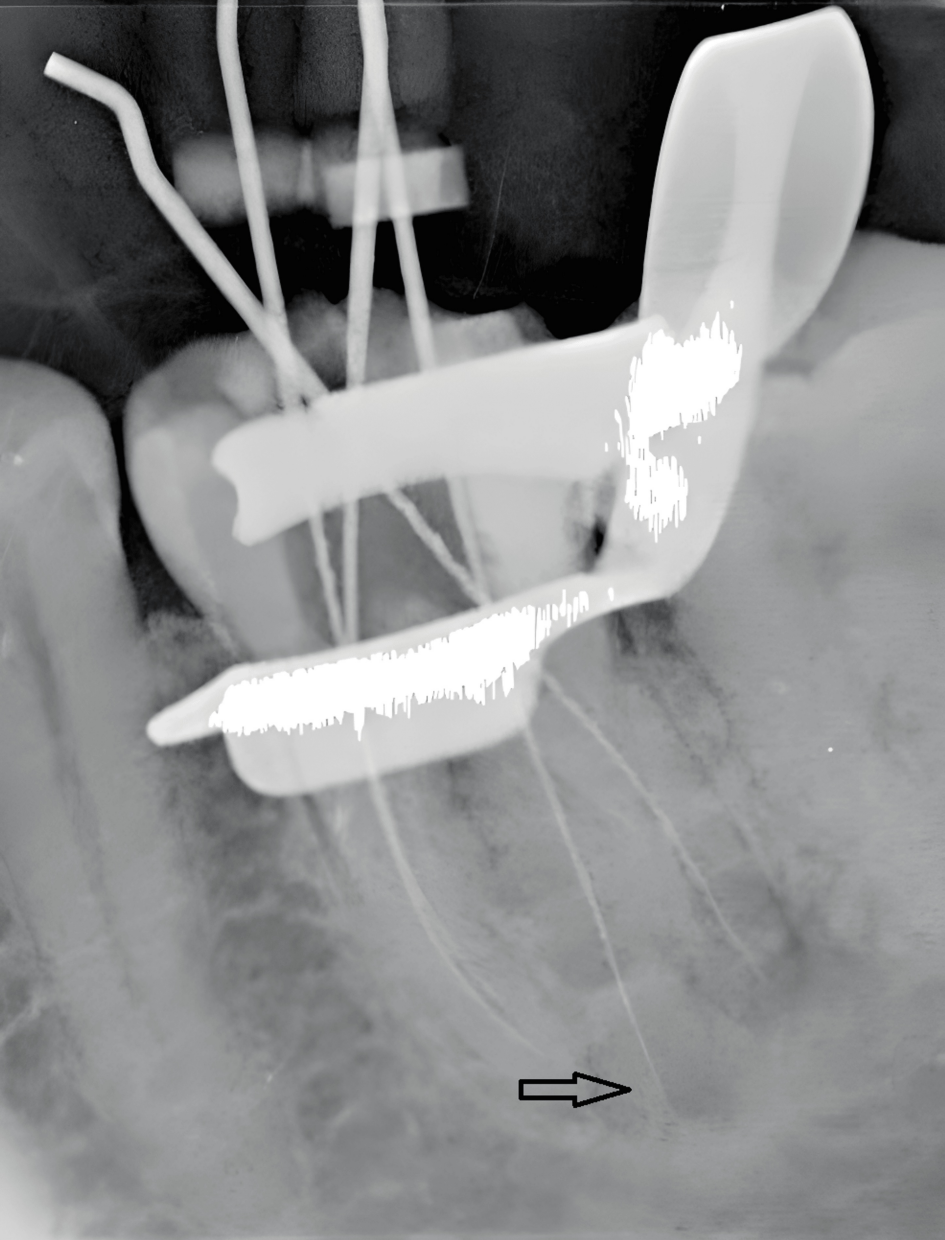
Working length radiograph of four canals.

Canal orifices were located using a Rhein endodontic explorer, and canal patency was confirmed using size #08, #10, and #15 K-files (Micro-Mega, Besançon, France). Working lengths were established radiographically (Figure 2). Biomechanical preparation was performed using the crown-down technique using the iRace rotary system (FKG Dentaire, La Chaux-de-Fonds, Switzerland).

**Figure 3:**
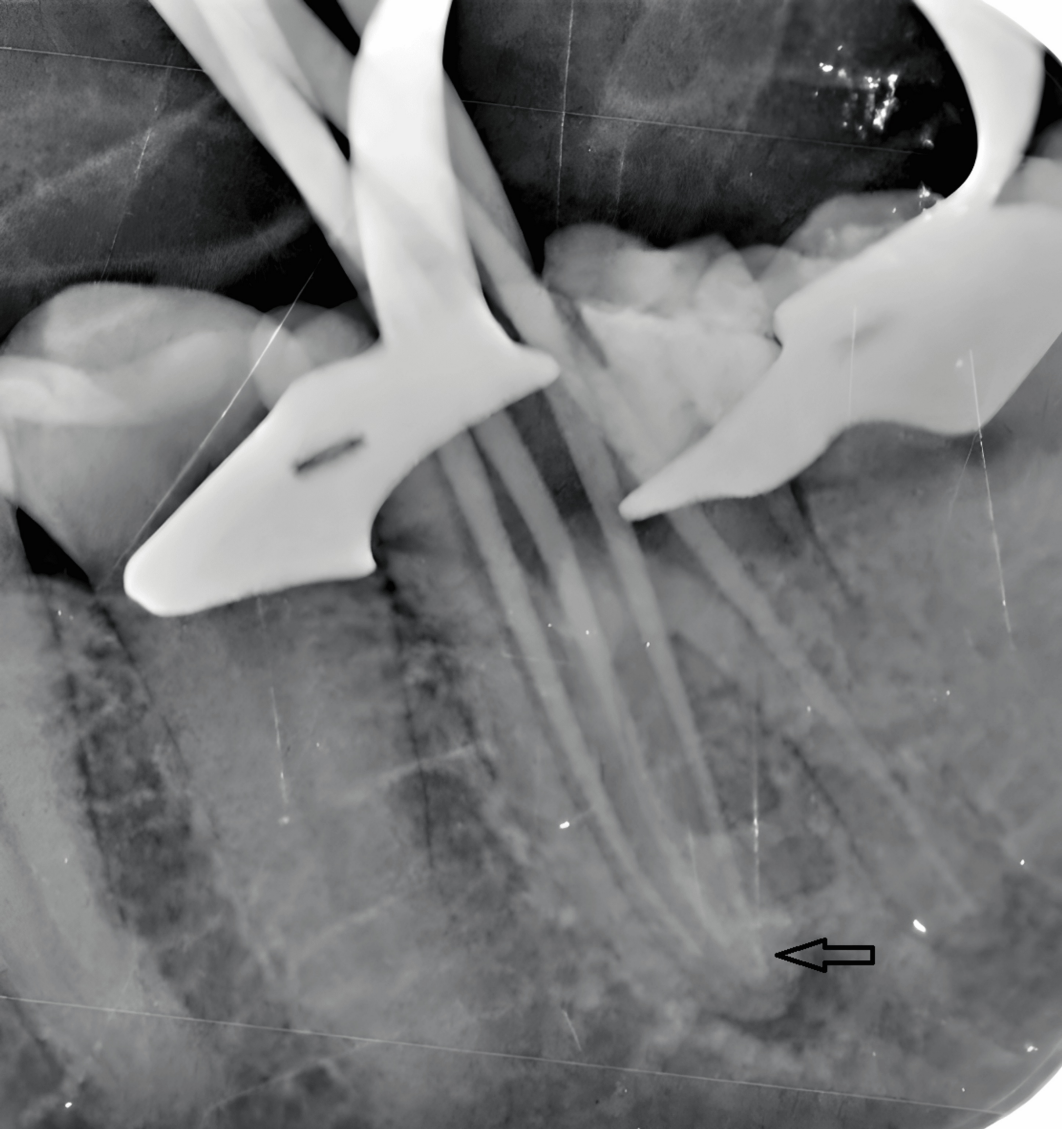
Master cone fit verification radiograph.

During canal preparation, 2 mL of 2.5% sodium hypochlorite (NaOCl) was used after the use of each instrument. For the final irrigation sequence, NaOCl was first neutralized with 5 mL of distilled water, followed by 5 mL of 17% ethylenediaminetetraacetic acid solution (Produits Dentaires SA, Switzerland), applied for one minute, and activated using the master cone at working length minus 1 mm. This was followed by another rinse with 5 mL of distilled water, and a final irrigation with 10 mL of 2.5% NaOCl to complete the disinfection protocol. After cone fitting, a radiograph was obtained to verify the accuracy of master cone placement (Figure 3).

**Figure 4:**
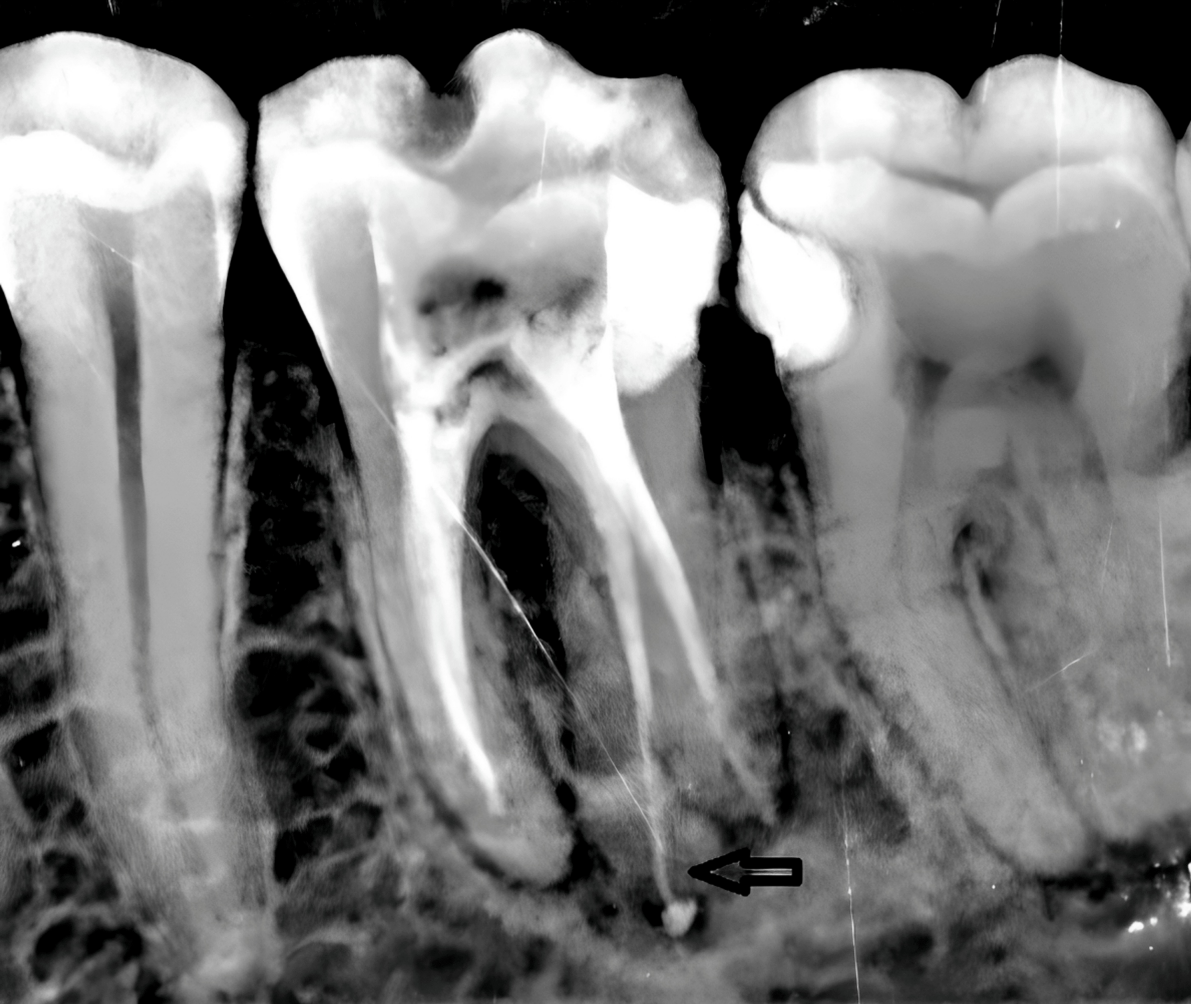
Post-obturation radiograph showing successful filling of four canals.

Root canal obturation was performed in a subsequent session using cold lateral condensation in the apical third and thermomechanical compaction in the coronal two-thirds, with gutta-percha points and the Revo-Condensor device (Micro-Mega, Besançon, France). An epoxy resin-based root canal sealer, ADSEAL (Meta Biomed Co., Ltd., Cheongju-si, South Korea), was used to ensure an effective seal of the root canal system (Figure 4).

The access cavity was restored using a glass-ionomer cement base (EQUIA, GC Corporation, Leuven, Belgium), followed by a laminated composite resin restoration (GRADIA, GC Corporation, Leuven, Belgium).

## Discussion

The primary objectives of root canal therapy are to thoroughly clean and shape the root canal system, followed by three-dimensional obturation to prevent reinfection [7]. Achieving successful endodontic outcomes requires a thorough understanding of root canal anatomy and its potential variations. One such variation is the RE, an additional distolingual root most commonly observed in mandibular molars. While it is relatively rare among Caucasian, African, and Indian populations (prevalence <5%), it occurs more frequently in populations with Asian traits, with reported rates ranging from 5% to 30%, depending on the population and study method used [4-6].

De Moor et al. (2004) [8] classified the RE as a short distolingual root that may exhibit a buccal curvature. However, in the present case, the RE observed did not conform to this morphological description as it was longer than the distobuccal root and lacked the typical buccal curvature. Chen et al. (2009) [6] also investigated the curvature of the RE, reporting a severe curvature (greater than 25°) in 90.5% of cases on mesiodistal radiographs. In contrast, the RE in our case showed no apparent mesiodistal curvature. The absence of a cone-beam computed tomography (CBCT) scan limits the ability to assess any buccolingual curvature. Based on Song et al.’s (2010) [9] CBCT-based classification, this case corresponds to a Type I morphology.

Preoperative analysis is essential in endodontics, especially when anatomical variations are suspected. The external morphology of three-rooted mandibular molars can be more complex than that of two-rooted teeth, and, in some cases, periodontal attachment loss and deeper probing depths may be observed in the area of the additional root [10]. CBCT can help overcome limitations associated with conventional radiographs by allowing three-dimensional visualization of complex root anatomies. It is especially valuable in retreatment cases, or when conventional radiographs fail to provide sufficient anatomical details. However, its use should be weighed against radiation exposure and cost, following the ALARA principle (As Low As Reasonably Achievable) [11].

Clinically, the presence of an RE is confirmed during access cavity preparation. To locate the fourth canal, the access cavity can be extended distolingually, resulting in a trapezoidal shape that facilitates visualization. The law of symmetry may assist in locating canal orifices, especially when the distobuccal canal is shifted buccally [12]. Other clinical signs include bleeding points, champagne bubble effect indicating the presence of organic pulp tissue, and visual cues such as developmental lines on the pulp chamber floor when viewed under magnification.

The presence of an RE has significant clinical implications. Instrumentation must be approached with caution to avoid perforation, particularly in cases with severely curved roots. Excessive shaping can weaken the root structure or cause ledging or instrument separation. It is advisable to maintain a conservative taper of approximately 4% to ensure adequate disinfection and sealing without compromising root integrity. During obturation, thermoplasticized techniques are preferred to minimize vertical root fracture risk and to better adapt to complex canal morphology. Caution is also warranted when preparing the canal for post placement, as over-preparation can further weaken the root or lead to perforation.

## Conclusions

Although the prevalence of RE is relatively low in most populations, its occurrence is significantly higher in individuals with Asian traits. Given the anatomical complexity and potential for severe curvature in such cases, clinicians should maintain a high index of suspicion and adapt their treatment strategies according to the endodontic anatomy of mandibular molars. A thorough preoperative assessment, careful access preparation, and an appropriate shaping and obturation strategy are essential to avoid iatrogenic complications. By recognizing and appropriately managing RE, clinicians can significantly improve endodontic outcomes and ensure long-term tooth preservation.
